# Coronary artery rupture in blunt thoracic trauma: a case report and review of literature

**DOI:** 10.1186/s13019-016-0528-6

**Published:** 2016-08-02

**Authors:** Jareer Heider Abu-Hmeidan, Arief Ismael Arrowaili, Raid Said Yousef, Sami Alasmari, Yasser M Kassim, Hamad Hamad Aldakhil Allah, Abdullah Mohammed Aljenaidel, Abdullah Abdulmohsen Alabdulqader, Muath Hamad Alrashed, Mulfi Ibrahim Alkhinjar, Nawwaf Rahi Al-Shammari

**Affiliations:** 1 Department of General Surgery, Prince Mohammed bin Abdulaziz Hospital, Al Imam Ahmad Ibn Hanbal Street, Ar Rawabi, Riyadh, 11676 Saudi Arabia; 2 Department of General Surgery, Al-Imam Muhammad Ibn Saud Islamic University School of Medicine, Airport Road, Riyadh, 13318 Saudi Arabia; 3 Department of Cardiology, Prince Mohammed bin Abdulaziz Hospital, Al Imam Ahmad Ibn Hanbal Street, Ar Rawabi, Riyadh, 11676 Saudi Arabia; 4 Al-Imam Muhammad Ibn Saud Islamic University School of Medicine, Airport Road, Riyadh, 13318 Saudi Arabia; 5 School of Medicine and Public Health, University of Newcastle, Callaghan, NSW 2308 Australia

**Keywords:** Coronary artery rupture, Blunt trauma, Thoracic trauma, Left anterior descending (LAD) coronary artery, Case report

## Abstract

**Background:**

Blunt thoracic trauma can rarely result in coronary artery injury. Blunt trauma can result in occlusion of any of the coronary arteries or can lead to its rupture and bleeding. Traumatic coronary artery occlusion can lead to myocardial infarction, while its rupture and bleeding can result in hemopericardium and cardiac tamponade, and can be rapidly fatal. Survival after coronary artery rupture in blunt thoracic trauma is exceedingly rare.

**Case Presentation:**

We present a case of a young male who sustained a blunt thoracic trauma in a motor vehicle collision, that resulted in rupture of the left anterior descending (LAD) coronary artery and subsequent cardiac tamponade. Prompt surgical intervention with pericardiotomy and ligation of the artery has resulted in survival of the patient.

**Conclusions:**

In cases of traumatic coronary artery rupture, early surgical intervention is crucial to avoid mortality. Ligation of the injured coronary is a viable option in selected cases, and can be the most expeditious option in patients in extremis.

## Background

Coronary artery injury in blunt chest trauma is uncommon yet potentially fatal. Autopsy studies reveal an incidence of around 2 % of all cardiac injuries caused by blunt chest trauma [1, 2]. The mechanical energy from trauma transmitted to coronaries can result in intimal tears, wall dissection, dislodgement or rupture of a pre-existing atherosclerotic plaque, coronary artery spasm, or coronary artery rupture [1, 3–5]. Fractured ribs from blunt trauma can result in coronary laceration [6, 7]. Lesions that result in subsequent occlusion of the involved coronary artery (like intimal tears, wall dissection, rupture of a pre-existing atheromatous plaque, and coronary artery spasm) can culminate in myocardial infarction, which can be immediate or delayed [4]. On the other hand, coronary rupture can result in precipitous development of hemopericardium and cardiac tamponade, and is thought to be universally fatal [1].

Few case reports exist on coronary artery rupture in blunt chest trauma. In cases were the pericardium remains intact, rapid development of cardiac tamponade makes survival improbable after such injuries. We present a rare case of left anterior descending (LAD) coronary artery rupture in a young male following blunt chest trauma in a motor vehicle collision. The patient survived despite developing cardiac tamponade and circulatory arrest, and was treated by emergency thoracotomy and ligation of the distal LAD.

## Case presentation

A 26-year-old male, who was not known to have any risk factors for coronary artery disease, presented to the emergency department after a head-on motor vehicle collision as unrestrained driver. The patient complained of chest pain and shortness of breath. His GCS was 15/15. He was tachypneic (RR: 45 breaths/min) and tachycardic (HR: 134 beats/min). He was found to be hypotensive upon arrival (BP: 87/54 mmHg), but his blood pressure normalized following the rapid infusion of two liters of lactated Ringer’s solution. Auscultation of lung fields revealed reduced breath sounds on the right side. Chest x-ray (CXR) revealed a large right-sided hemothorax. A thoracostomy tube was inserted on the right side resulting in the immediate drainage of two liters of blood. Initial focused assessment with sonography in trauma (FAST) exam was negative, but the patient became hypotensive again soon after (BP: 60/40 mmHg). Repeated FAST revealed a moderate amount of fluid in the pericardial space. The patient was rushed into the operating room for thoracotomy and pericardiotomy. Induction of anesthesia was accompanied by progressive bradycardia that degenerated rapidly into asystole. A resuscitative thoracotomy (left anterolateral) revealed hemopericardium and cardiac tamponade. The pericardium was intact. Pericardiotomy and evacuation of more than 200 ml of blood from the pericardium, followed by internal cardiac massage, resulted in restoration of heart activity after 3 min. With restoration of spontaneous circulation, bleeding was noticed from a small perforation in the distal LAD near the apex. The distal LAD was ligated proximal to the site of perforation. The left anterolateral thoracotomy was extended across the midline to the right side into a clamshell thoracotomy to allow for proper exploration of the right hemithorax. The bleeding was found to arise from multiple injured intercostal vessels, and hemostasis was secured. The blood pressure started to improve, and the patient was sent to the intensive care unit on mechanical ventilation.

Cardiac markers were all elevated at presentation (CK: 4,267 U/L, Ref. Range 30–200. CK-MB: 226 U/L, Ref. Range <25 U/L. Troponin I: 19.29 ng/ml, Ref. Range 0.04 ng/ml), and continued to rise to reach a peak at 48 h (CK: 32,109 U/L. CK-MB: >1000 U/L. Troponin I: 19.43 ng/ml), after which they started to decline. A standard 12 lead ECG showed sinus tachycardia with left axis deviation, and a down sloping ST segment depression of anterolateral leads (V1-V6) with T wave inversion. A transthoracic echocardiogram (TTE) was done on the first postoperative day. It showed a normal left ventricular (LV) function with an ejection fraction (EF) of 60 %. The echocardiogram did not show any wall motion abnormalities at that point, and there was no significant valvular pathology.

There was considerable difficulty in liberating the patient from mechanical ventilation due to underlying pulmonary edema and bilateral lung contusions. The patient was extubated after 14 days of mechanical ventilation. A follow up TTE done 4 weeks later showed a moderate LV systolic dysfunction with an EF of 35 %, and wall motion abnormalities involving the apical and anterolateral regions. The patient was discharged from hospital few days later on anti-heart failure medication.

## Discussion

Albeit rare, blunt chest trauma may result in the occlusion of any of the coronary arteries or may lead to its rupture and bleeding. Each of these two patterns of injury is associated with a distinct corresponding patient population, clinical presentation, management, and outcome.

Intimal tears, wall dissection, disruption of atherosclerotic plaque, coronary artery spasm, and epicardial hematomas can result in partial or complete occlusion of the coronary artery and subsequent myocardial ischemia. Depending on the severity of the occlusion and the rapidity by which it develops, patients may present with angina pectoris, myocardial infarction, or even heart failure many years after the injury. Christensen et al. conducted a review on cases of myocardial ischemia following blunt chest trauma [8]. The review showed that 82 % of the 77 studied patients were below the age of 45. All of the reported patients developed myocardial infarction due to occlusive lesions of the coronary arteries, except for one patient who had angina pectoris without evidence of infarction. The LAD was the most common coronary involved. Only 5 of the 77 (6.5 %) reported cases died secondary to the coronary artery injury and ischemia. The favorable prognosis was attributed to the patients’ young age, and to the involvement of a single vessel. Percutaneous transluminal coronary angioplasty (PTCA) and stenting is thought to be the most suitable treatment in these cases [8].

Rupture or laceration of a coronary artery can result in bleeding into the pericardial or pleural cavities. Aside from laceration caused by a broken rib [6, 7], the mechanism by which a coronary artery ruptures is not well understood. Shearing forces from craniocaudal deceleration, and compression of the chest while the patient is holding his breath (compression-Valsalva injury) have been implicated as possible mechanisms [9]. Sudden increase in the transmural pressure of the coronary artery, resulting from compression of the heart between the sternum and spine, might lead to rupture when the increase in pressure exceeds the compliance of the artery. Atherosclerosis results in decreased elasticity of the coronary artery, and turns it into a rigid tube that is more liable to rupture with sudden increases in transmural pressure [5, 10, 11]. This helps to explain why most of the reported cases of coronary artery rupture involve older patients with pre-existing coronary artery disease, which is in contrast to the patient population observed in cases of coronary occlusion, where the majority are below the age of 45 and do not have a pre-existing coronary artery disease. The right coronary artery (RCA), at or near its origin, is most commonly involved [Table 1]. The accumulation of blood in the closed pericardial space results in the precipitous development of cardiac tamponade and consequent shock. However, bleeding can occur into the pleural cavity through a rent in the pericardium. This prevents the rapid development of cardiac tamponade [11], allowing a longer time window before the development of shock and subsequent mortality. In contrast to cases of coronary occlusion discussed above where mortality is due to myocardial ischemia and infarction, hemopericardium and cardiac tamponade are the important sequela of coronary laceration that contribute to mortality [1]. Injury to other cardiac structures or great vessels in association with the coronary artery injury can increase mortality. Advanced age and pre-existing coronary artery disease can also play a role in the worse outcome observed in patients with this pattern of injury when compared to patients with traumatic coronary occlusion.

**Table 1:** Summary of reported cases of coronary artery rupture in blunt thoracic trauma

Reference	Age & gender	Mechanism of injury	Diagnosis	Presentation	Artery involved	Associated cardiac injuries	Pericardial defect	Coronary artery disease	Management	Time window^a^	Outcome
Goffin et al. 1974 [11].	62 M	MVC	Autopsy	Dyspnea, chest pain, shock. CXR: Multiple rib fractures. Slight enlargement of the heart. ECG changes of inf. MI and AV block. Moderately elevated CPK and SGOT.	RCA	Aortic tear. Intimal tear in LCX	Present	Present	Pericardiocentesis (failed)	9.5 h	Died
Trotter et al. 1998 [9].	15 M	MVC	Intraop.	Chest wall contusion. Continuous murmur. Initially stable vital signs, then worsening dyspnea and cardiogenic shock. CXR: widened cardiac silhouette. RBB on ECG.	RCA	Tricuspid valve	Present	N/A^b^	ACB	8 h	Survived
Suzuki et al. 2000 [6].	59 M	FD	Autopsy	Severe dyspnea and chest pain 14 h after the incident. CXR: fractures of left 7th and 8th ribs with left pleural effusion.	LAD	None	Present	N/A^b^	None	17 h	Died
Dimopoulos et al. 2003 [16].	78 F	FD	Autopsy	Anemia. Massive left pleural effusion on CXR. Atrial fibrillation and LBBB. Echocardiogram: 700 ml of pericardial effusion with normal ejection fraction.	ICA	None	N/A^b^	N/A^b^	None	N/A	Died
Straub et al. 2003 [10].	71 M	FD	Intraop.	No visible chest wall lesions. Cardiogenic shock. CXR: normal. Elevated cardiac enzymes. Echocardiography: pericardial effusion, no wall motion disturbances. CT scan: hemorrhagic pericardial effusion and para-aortic contrast fluid extravasation.	RCA	None	None	Present	OPCAB	14 h	Died
Sugimoto et al. 2003 [17].	34 M	MVC	Intraop.	Unconscious and in severe shock. Echocardiogram: massive hemopericardium.	RCA	None	N/A^b^	N/A^b^	ER thoracotomy with attempted ligation of the RCA. Interval ACB three weeks later.	No delay	Survived
Dueholm et al. 2009 [12].	44 M	MVC	Autopsy	Chest pain. Normal exam. ECG: Acute MI. CXR: Fractures in left 6th and 7th ribs. Arrhythmia, followed by cardiogenic shock.	RCA	None	N/A^b^	Present	None.	56 h	Died
Tyson et al. 2010 [7].	69 M	MVC	Intraop.	Breath sounds decreased on the left. CXR: left hemothorax and widened mediastinum. CT scan: minimal pericardial fluid. Fractures in left 2nd -7th ribs. Tube thoracostomy drained 2 L of blood.	OM	Posterior cardiac vein	Present	N/A^b^	Anterolateral thoracotomy and ligation of bleeding vessels.	2 h	Survived
Burcar et al. 2013 [18].	15 M	MVC	Intraop.	Chest wall hematomas. GCS 5/15. Hypotension. ST segment depression. Troponin I & CPK elevation. Bilateral lung contusions on CT scan.	LM	Intimal tear in MPA	N/A^b^	N/A^b^	Sternotomy. Pericardiotomy. LIMA to LAD. SVG to LCX.	N/A	Died

*MVC* motor vehicle collision, *FD* falling down, *LAD* left anterior descending artery, *RCA* right coronary artery, *LCX* circumflex artery, *ICM* intermediate coronary artery, *OM* obtuse marginal artery, *LM* left main coronary artery, *MPA* main pulmonary artery, *ACB* aortocoronary bypass, *OPCAB* off pump coronary artery bypass, *LIMA* left internal mammary artery, *SVG* saphenous vein graft

^a^Time window between injury and intervention or death

^b^No explicit information about the finding was provided in the case report

Unlike cases of coronary occlusion that has been reported after apparently trivial trauma, the amount of energy needed to rupture a coronary artery is high and is usually associated with high-speed motor vehicle collisions. The clinical picture is variable and patients may complain of chest pain and might be hemodynamically unstable. The injury is rarely suspected and the clinical picture is usually attributed to more common causes of chest pain in blunt chest trauma. ECG changes are non-specific and are usually attributed, along with elevated cardiac enzymes, to cardiac contusion. CXR may show fractured ribs but coronary artery rupture may occur with an intact thoracic skeleton [12]. The heart shadow may appear enlarged due to hemopericardium, but a traumatic communication between the pericardial and pleural cavities can result in the appearance of pleural effusion in its place [6, 7]. Similarly, pericardial effusion, which is the principal manifestation of coronary artery rupture, can be undetectable or absent on FAST exam if the pericardium is not intact. In addition, bleeding from the ruptured coronary can seep beneath the epicardium and become contained in the form of an epicardial hematoma that could be missed on FAST examination or mistaken for chamber dilatation. This phenomenon can also result in failed attempts at pericardiocentesis or subxiphoid pericardiotomy [10]. Rarely, spasm in the severed coronary artery can result in absence of pericardial effusion despite an intact pericardium [12].

Contrary to popular belief that such injuries are usually instantly fatal, accounts of coronary artery rupture report a time window ranging from 2 to 56 h [Table 1]. This time window could theoretically be sufficient to avoid mortality when timely management is provided. Patients with pericardial effusion or hemodynamic instability should undergo prompt exploratory surgery. As discussed above, pericardiocentesis might be of limited utility in these cases since it has been observed that blood can accumulate in the form of epicardial hematoma instead of collecting in the pericardial space. In addition, despite the presence of pericardial effusion, coronary injury was only identified intraoperatively or at autopsy and was not suspected preoperatively in most reported cases [Table 1]. Therefore, a low threshold for timely thoracotomy and exploration should be kept in patients with blunt chest trauma and pericardial effusion or hemodynamic instability. More stable patients, in absence of pericardial effusion, who continue to complain of chest pain or other cardiac symptoms, and those who show evidence of myocardial ischemia on electrocardiogram or elevated cardiac enzymes, can undergo formal echocardiogram followed by coronary angiogram to detect any coronary injury.

Operative options in the management of coronary artery rupture include ligation or revascularization. The choice of incision is dictated by the clinical situation. A left anterolateral thoracotomy incision is used in patients who need an emergency department or resuscitative thoracotomy, while median sternotomy is preferred in the more stable patient. The left anterolateral incision can be extended across the midline to the right side into a clamshell thoracotomy for better exposure if needed. The decision to ligate or revascularize the injured coronary artery depends on the amount of ventricular mass that would be affected by the interruption of the artery, and on the physiologic status of the patient. Distal injuries in main coronary arteries, and most injuries in secondary coronary arteries such as the diagonal, obtuse marginal, or acute marginal coronary arteries, can be treated by ligation. Ligation in such injuries might result in a small area of infarction that does not usually lead to significant cardiac dysfunction [13]. In contrast, ligation of proximal injuries in main coronary arteries might result in a widespread infarct, with consequent intractable heart failure and fatal arrhythmias. Revascularization is typically indicated in these injuries. However, it is theorized that the majority of patients with significant proximal coronary artery injuries suffer widespread cardiac ischemia, fibrillate, and die earlier in their course of injury, such that survival to care is an implicit indicator that ligating the injured coronary artery is likely to be tolerated by the patient [13]. In patients with severe physiologic derangements and a proximal coronary artery injury, who are unlikely to tolerate an immediate revascularization procedure, it is appropriate to use a damage control approach by ligating the artery, resuscitating and rewarming the patient, correcting coagulopathy, and performing revascularization on a later date if needed. Intra-aortic balloon pump can be used as a temporary measure in patients who develop cardiac dysfunction after coronary artery ligation [13].

**Fig. 1:**
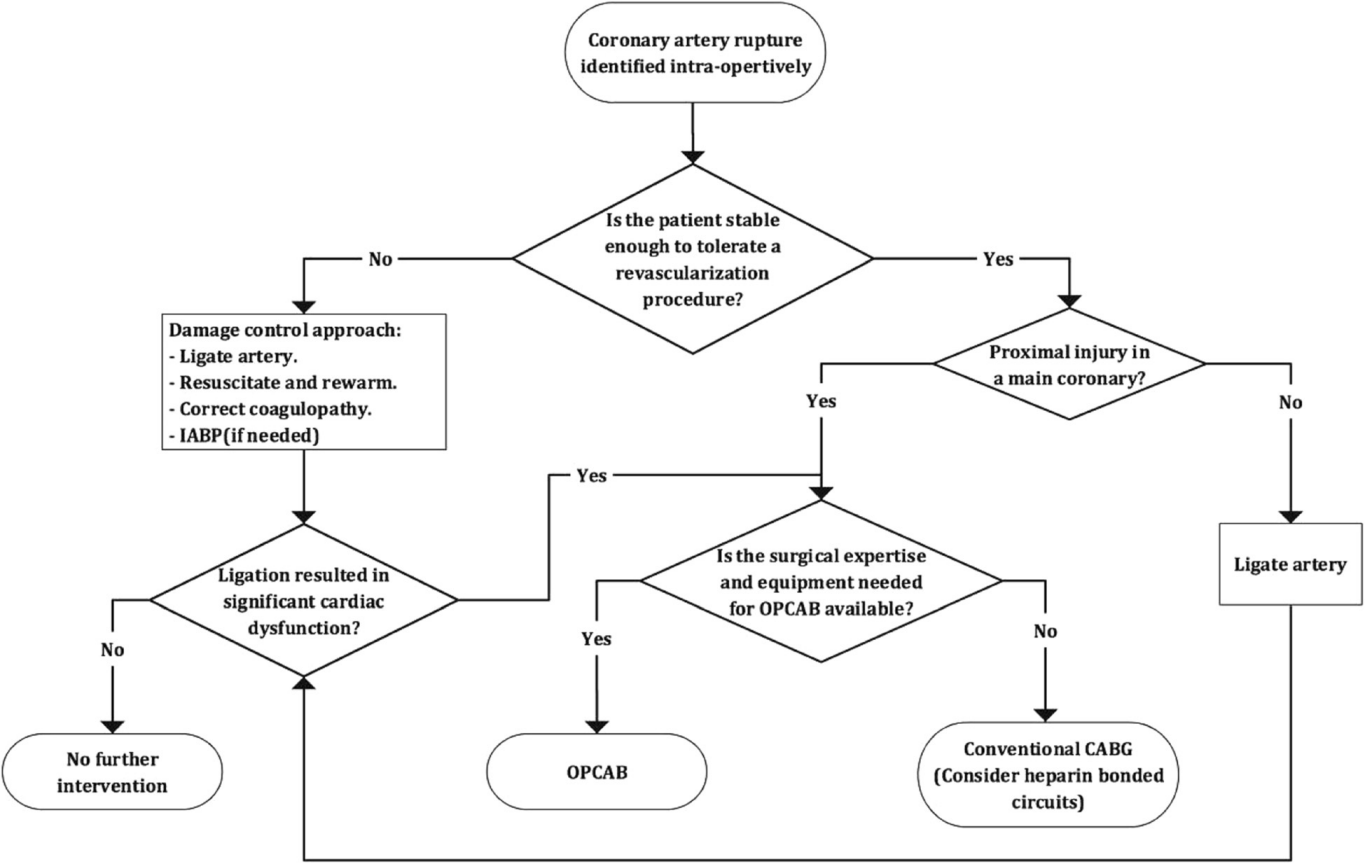
A proposed algorithm for the management of a ruptured coronary artery identified intra-operatively in a multi-trauma patient. CABG: coronary artery bypass grafting. IABP: intra-aortic balloon pump. OPCAB: off-pump coronary artery bypass

Cardiopulmonary bypass (CPB) has traditionally been used for coronary revascularization in the acute setting. Full systemic heparinization required for the extracorporeal circulation in CPB can be hazardous in patients with multiple injuries or suspected central nervous system injury. The use of off –pump coronary artery bypass grafting (OPCAB) can reduce coagulopathy and transfusion requirement, and have the added benefit of attenuated inflammatory response in comparison with conventional, on-pump, coronary artery bypass grafting (CABG) [14]. These benefits are of particular importance in trauma patients. Nevertheless, OPCAB entails performing a vascular anastomosis on a beating heart, which requires specialized cardiovascular surgical skills and familiarity with the different mechanical stabilizers used in the procedure. These requirements might be an issue in the acute setting of trauma. The on-pump beating heart procedure poses the same requirements as OPCAB in terms of specialized skills and availability of special equipment, yet have the downside of an extracorporeal circuit, making the value of this procedure questionable in the trauma patient. Heparin bonded circuits have been shown to decrease the incidence of postoperative blood transfusion (but not the postoperative blood loss) following open heart surgery [15], and could be of some value in trauma patients. In the rare instance when a partial coronary artery rupture is identified preoperatively, coronary angiogram and stenting over the rupture can be utilized in stable patients. [Figure 1] shows a proposed algorithm for the management of coronary artery rupture identified intra-operatively in the multi-trauma patient. It is important to keep in mind that the lack of large studies looking into the different lines of treatment in this area makes definitive statements on the most appropriate medical intervention difficult to make.

## Conclusion

Coronary artery rupture is a rare occurrence in blunt trauma, and high index of suspicion is needed to identify these injuries. Low threshold for exploratory surgery should be kept in patients manifesting pericardial effusion or hemodynamic instability. These injuries are compatible with a short period of survival, and may be survivable with proper resuscitation and timely surgery.

Many of these injuries can be safely treated by ligation.

## Abbreviations

ACB, aortocoronary bypass; CABG, coronary artery bypass grafting; CXR, chest x-ray; EF, ejection fraction; FAST, focused assessment with sonography in trauma; FD, falling down; IABP, intra-aortic balloon pump; ICM, intermediate coronary artery; LAD, left anterior descending artery; LCX, circumflex artery; LIMA, left internal mammary artery; LM, left main coronary artery; LV, left ventricular; MPA, main pulmonary artery; MVC, motor vehicle collision; OM, obtuse marginal artery; OPCAB, off –pump coronary artery bypass; PTCA, percutaneous transluminal coronary angioplasty; RCA, right coronary artery; SVG, saphenous vein graft; TTE, transthoracic echocardiogram
